# *Neat1* in hematopoietic stem cells

**DOI:** 10.18632/oncotarget.22729

**Published:** 2017-11-30

**Authors:** Noam Fallik, Yael Bar-Lavan, Yariv Greenshpan, Oron Goldstein, Markus Grosch, Micha Drukker, Roi Gazit

**Affiliations:** ^1^ The Shraga Segal Department for Microbiology Immunology and Genetics, Faculty of Health Sciences, The Ben-Gurion University of the Negev, Be’er Sheva, Israel; ^2^ National Institute for Biotechnology in the Negev, The Ben-Gurion University of the Negev, Be’er Sheva, Israel; ^3^ Center for Regenerative Medicine and Stem Cells, The Ben-Gurion University of the Negev, Be’er Sheva, Israel; ^4^ Institute of Stem Cell Research, German Research Center for Environmental Health, Helmholtz Center Munich, Neuherberg, Germany

**Keywords:** *Neat1*, hematopoietic stem cells, paraspeckles, hematopoiesis, long non-coding RNAs

## Abstract

Hematopoietic Stem Cells (HSCs) generate blood and immune cells through a hierarchical process of differentiation. Genes that regulate this process are of great interest for understanding normal and also malignant hematopoiesis. Surprisingly, however, very little is known about long-non-coding RNAs (lncRNA) in HSCs. *Neat1* is a lncRNA that plays a major role in the formation of sub-nuclear structures called paraspeckles, and was reported to regulate proliferation and differentiation in other cells types. We detected *Neat1* expression using RNA-seq data and RT-qPCR in HSCs, progenitors and effector immune cells, by specific detection of its isoforms. *Neat1* is highly expressed in stem and progenitor cells, yet it shows significant reduction in granulocytes. Microscopically, *Neat1* is detected as sharp nuclear foci. Paraspeckle proteins NONO and PSPC1 are detected as aggregated nuclear foci in fresh primary hematopoietic cells, and in cultured cells. Induction of differentiation *in vitro* was found to enhance *Neat1* expression. Taken together, our data demonstrate for the first time the expression of *Neat1* and paraspeckles formation in HSCs and along hematopoiesis.

## INTRODUCTION

The identity of a cell is determined by the genes it expresses. Protein-coding genes had been the focus of research in the past, in recent years the importance of non-coding genes has also been reported to differentially express and play a role in processes such as differentiation, normal cell state maintenance and malignancy [1-5]. The hematopoietic system consists of well-characterized cell types from the multipotent hematopoietic stem cells (HSC) through defined progenitors and down to the various effector cells. Studying non-coding genes in normal and perturbed hematopoiesis is of interest in order to reveal new insights into this essential process of blood cell generation, and possibly related malignancies. Specific studies of non-coding genes of interest are needed to further characterize their expression along hematopoiesis.

Nuclear Enriched Abundant Transcript 1 (*Neat1*) is a long non-coding RNA (lncRNA) that was cloned as polyadenylated nuclear enriched RNA [6]. *Neat1* has two isoforms: *Neat1_1* and *Neat1_2* also known as Multiple Endocrine Neoplasia ε and β (*Menε* and *Menβ*, respectively) [1]. These two isoforms are transcribed from a single locus of chromosome 19 in mice or 11 in humans, with a difference in their 3’ ends [6, 7]. *Neat1_1* is a 3170 bp polyadenylated transcript, whereas *Neat1_2* is a 20,177 bp transcript containing a genomic poly(A)-rich tract on its 3’ end [1, 8-10]. Both isoforms were reported to play an essential role as the core of the sub-nuclear structures called paraspeckles [11]. Intriguingly, it is not yet clear which of the isoforms is more significant in maintaining paraspeckle integrity, due to the fact that they are produced from a single locus. Moreover, it is difficult to stop the transcription of *Neat1_1* without affecting *Neat1_2*, although vice versa might be possible. Thus, both *Neat1_1* and *Neat1_2* are considered as core-components of the paraspeckles, and are of interest for specific studies on different cell types [1, 11].

Paraspeckles are sub-nuclear structures composed of functionally distinct proteins including PSPC1, SFPQ and NONO (also known as p54^nrb^), that are in a complex together with *Neat1* [8, 12, 13]. Paraspeckles play a role in the regulation of certain genes in differentiated cells by nuclear retention of RNA, controlling gene expression by trapping adenosine to inosine (A to I) hyper-edited RNA within the nucleus [14, 15]. This mechanism of mRNA retention can be used to coordinate gene expression by release upon need, such as stress [12, 15]. Furthermore, a recent study discovered that NONO, PSF and *NEAT1* in HeLa cells mediate pri-miRNA processing, with a structural role for NEAT1_2 isoform in recruitment of miRNA microprocessors, highlighting potential role in broad regulation of gene expression [16]. The physiological role of *Neat1* is not yet known, as viability under normal conditions was not severely affected in knockout mice [11], while the paraspeckle proteins were distributed across the nucleoplasm and the number of paraspeckle foci was decreased in *Neat1* deficiency [10]. Interestingly, it was shown that upon infliction of stress such as Polyinosinic:polycytidylic acid induction of type-I interferon response, there was an increase in the transcription levels of *Neat1* and in the formation of paraspeckle foci in the nucleus [4, 9, 10, 17]. Neither *Neat1* nor the paraspeckles proteins were studied so far in normal or perturbed hematopoiesis.

Paraspeckles are found in almost every cell type, including primary cells and cell lines, except for human embryonic stem cells (hESC) [11]. Interestingly, when hESC were differentiated in culture, *Neat1* was upregulated and paraspeckle foci were demonstrated to form in non-pluripotent cells [11]. Furthermore, recent studies suggested that the lack of expression of *Neat1* and paraspeckles might indicate a loss of pluripotency in hESC [18]. Therefore, if a cell does not express *Neat1* and/or paraspeckles this might serve as a marker for loss of pluripotency [18]. Induction of *Neat1* and paraspeckles was also shown on the differentiation of myoblasts into myotubes, with a three-fold up-regulation of *Neat1* and an increase in paraspeckle number and size [1]. This is most significant with the recent discovery of *NEAT1* isoforms role in pri-miRNA processing [16]. Intriguingly, the role of *Neat1* and paraspeckles has not yet been studied in hematopoiesis before, although findings in other cell types make it an interesting topic for research in the context of adult stem cell and differentiation.

HSCs research is leading both basic research and clinical applications of adult stem cells [19]. Therefore, the finding of paraspeckles which hold the potential to influence proliferation and differentiation of HSCs is of great interest. Characterization of *Neat1* expression and its isoforms in primary HSCs and in defined hematopoietic progenitors is needed in order to establish its possible role in early hematopoiesis. Through the study of HSCs and the factors that influence their potential to proliferate and differentiate, we may achieve new innovations in the study of bone marrow transplantation and blood cancers [19]. Paraspeckles were reported to correlate with the cell cycle and are associated with the appearance of RNA polymerase II and changes in the metabolic activity of cells [8, 12]. These properties are of major interest in hematopoiesis, making *Neat1* and paraspeckles a focal point of interest for research in HSCs. Recently, it was reported that *Neat1* is induced by p53 and plays a role in suppressing transformation and cancer initiation [20], thus strongly suggesting *Neat1* as a new factor on early malignancy.

In this study, we analyzed the expression of *Neat1* lncRNA and of paraspeckle proteins in mouse HSCs and in their direct progeny. RNA-seq data and RT-qPCR show that *Neat1_1* is highly expressed in HSCs and in early progenitors, while *Neat1_2* is expressed at lower levels. FISH identified bright *Neat1* foci in the nucleus, and paraspeckle proteins NONO and PSPC1 show distinguished nuclear expression. As for differentiated hematopoietic cells, granulocytes have the lower expression levels of *Neat1*, NONO and PSPC1, while B-cells maintain relatively higher expression levels of all three. Furthermore, ex-vivo cultured cells presented increased expression of *Neat1* when differentiation was induced. Taken together, our data present multiple evidence for *Neat1* and paraspeckles in different stages of hematopoiesis for the first time.

## RESULTS

### *Neat1* is expressed throughout hematopoiesis

**Figure 1:**
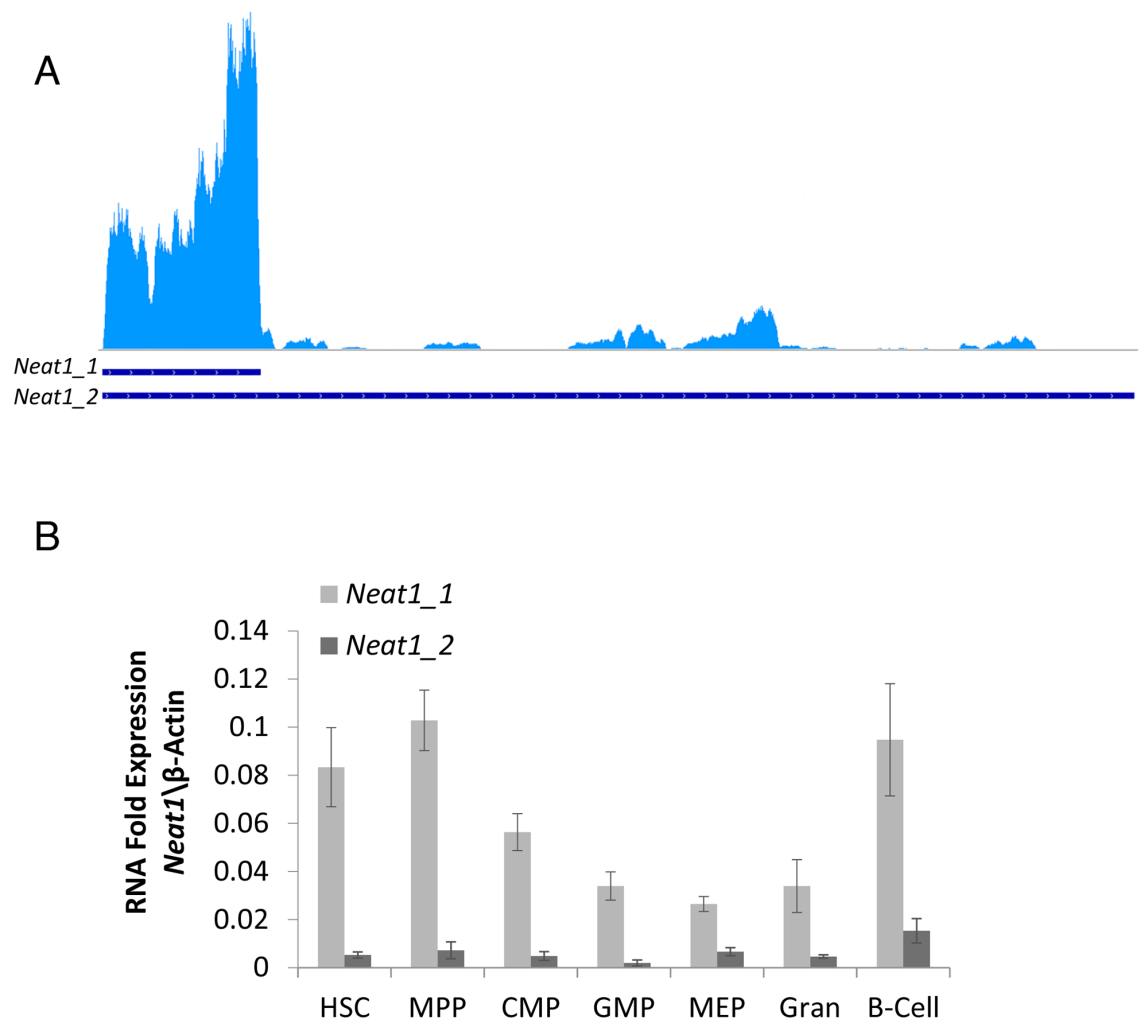
*Neat1_1* and *Neat1_2* RNA expression in hematopoietic stem cells **(A)** *Neat1_1* and *Neat1_2* lncRNA transcripts of 3170bp and 20177bp, respectively, presented by blue lines on bottom. RNA-seq data show that *Neat1_1* Is highly expressed in HSCs, whereas *Neat1_2* is expressed to a much lesser extent. **(B)** RT-qPCR detection of *Neat1_1* and *Neat1_2*, shown as relative to β-actin, from HSC, MPP, CMP, GMP, MEP, granulocyte, B-cells. Histograms show averages ±SD.

To begin the study of *Neat1* in HSCs, we first evaluated the specific expression levels of *Neat1_1* and *Neat1_2* by analyzing RNA-seq data [21]. The aligned-reads clearly showed that the *Neat1_1* isoform is highly expressed in HSCs (Figure 1A). RNA-seq data suggested that *Neat1_2* isoform was expressed to a much lesser extent, and there was no complete transcript of *Neat1_2* profoundly detected (Figure 1A, see longer-transcript). Since *Neat1* has a structural role, and the longer-isoform might be less well detected by the RNA-seq, we performed independent RT-qPCR for *Neat1_1* and *Neat 1_2* on mouse HSCs, progenitors and effector immune cells. HSCs, as well as other hematopoietic progenitors and differentiated cell types, showed a significant difference between *Neat1_1* to *Neat1_2* expression in primary cells (Figure 1B with HSC: *p<*0.01; MPP: *p<*0.01; CMP: *p<*0.01; GMP: *p<*0.01; MEP: *p<*0.01; Gran: *p<*0.01; B-Cells: *p=*0.018). Moreover, there was a significant difference in the levels of *Neat1*_1 expression between HSCs to GMPs (*p<*0.01), MEP (*p=*0.019), and granulocytes (*p=*0.012) in that HSCs had higher expression by 2.45, 3.12, and 2.52-fold, respectively. The rest of the cells examined (MPP, CMP and B-Cells) showed *Neat1_1* expression levels which did not significantly differ of HSCs (Figure 1B). Concerning the relative *Neat1_2* expression, HSCs showed higher expression levels compared to GMPs, by 3.09-fold (*p=*0.032). Notably, the RT-qPCR data suggests relatively high levels of *Neat1* in HSCs, at about 0.1-fold of the expression of β-Actin which is an extremely abundant transcript (Figure 1B).

### *Neat1* RNA forms sharp foci in HSCs and progenitors

**Figure 2:**
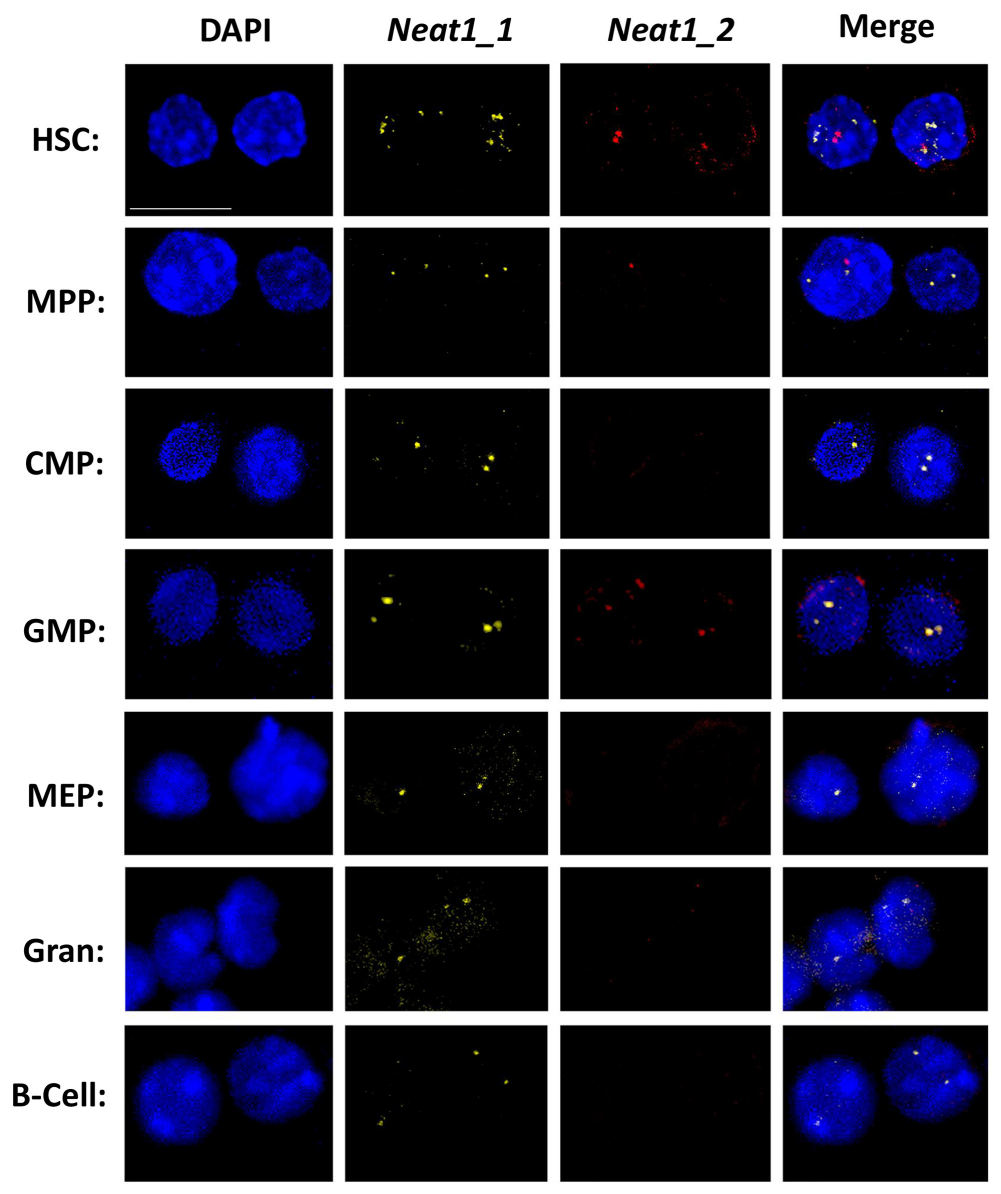
Primary NEAT RNA forms sharp foci in HSCs and progenitors Confocal microscopy analysis of *Neat1_1* and *Neat1_2* using FISH, in representative primary cell types through hematopoiesis, HSC, MPP, CMP, GMP, MEP, granulocyte, B cell. *Neat1_1* (yellow) and *Neat1_2* (red) are superimposed over nuclei stained with DAPI (blue). Scale bar denotes 10 μ.m.

After we managed to show RNA expression of *Neat1_1* and *Neat1_2*, we wanted to visualize it in HSCs, progenitors and differentiated cells. We used FISH technique aimed for *Neat1* isoforms, using commercial Stellaris® FISH Probes. The representative panels show that each primary cell type has usually 1 or 2 foci of *Neat1_1/2* which are all localized in the nucleus, and may suggest the formation of paraspeckles in these cells (Figure 2). Interestingly, when both *Neat1_1* and *Neat1_2* are detected they seem to colocalize as shown for example in HSCs and GMPs (Figure 2). This reinforces the existence of paraspeckles in mouse hematopoietic stem- and progenitor cells.

Regarding expression levels, *Neat1_1* was more vivid in all the cell types that we examined, in agreement with RNA-seq and RT-qPCR data (Figure 1). *Neat1_2* was bright only in some cells such as HSCs, but not easily detected in granulocytes (Figure 2). It seems that there is a slight agreement between the RT-qPCR data (Figure 1B) and the images acquired using FISH, possibly due to inherent differences between the techniques. Taken together, our data demonstrate the expression and the nuclear localization of *Neat1* in hematopoietic stem-cells, progenitors, and effector immune cells.

### Paraspeckle protein NONO displays aggregated clusters in hematopoietic cells

**Figure 3:**
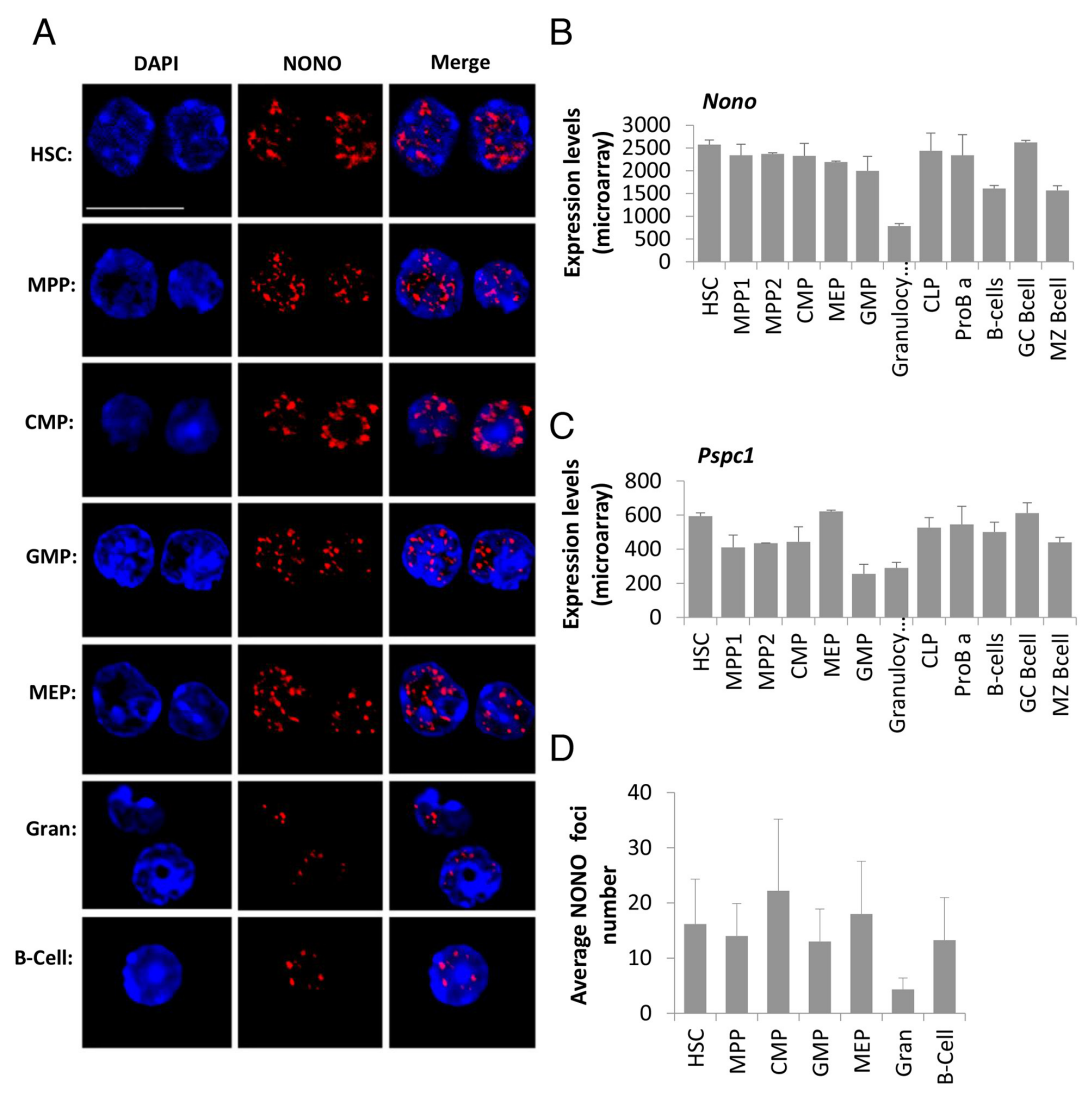
Paraspeckle protein NONO presents aggregated clusters in hematopoietic cells’ nuclei and reduced expression in granulocytes **(A)** Confocal microscopy analysis of NONO, a paraspeckle protein, using an immunofluorescent staining technique, in representative primary cell types through hematopoiesis, HSC, MPP, CMP, GMP, MEP, granulocyte, B cell. NONO (red) is superimposed over nuclei stained with DAPI (blue). Scale bar denotes 10 μ.m. **(B and C)** Expression levels of mRNA of the paraspeckles proteins NONO and PSPC1, respectively. Data is from ImmGen’s microarray database, showing averages of at least triplicates per cell type except for MPP and MEP that are duplicates; and SD per cell type. **(D)** Quantification of the foci number per cell type from immunofluorescent stained cells for NONO. Histograms show averages ±SD.

In addition to *Neat1* RNA, we wanted to visualize known paraspeckle proteins [12]. We visualized NONO and PSPC1 using immunofluorescence. Representative images show that each primary cell type tested expresses nuclear NONO foci with variance in amount, fluorescence intensity and size (Figure 3A). These foci may present paraspeckles which according to the literature can be in different size, number and intensity depending on cell type, and its cell cycle state [1, 7, 22]. Furthermore, the fact that they are seen in foci and not stained in a diffuse manner suggests that they might be attached to a paraspeckle. Interestingly, the visualized NONO-foci seem to localize mostly in the sub-areas of the nucleus that are more euchromatin, and there were no foci in dense DNA areas which are heterochromatin rich (Figure 3A and Supplementary Figure 1).

### Paraspeckles proteins show reduced expression in granulocytes

In order to quantify the paraspeckle proteins we first used published expression data from the ImmGen consortium [23] to determine the mRNA expression levels of *Nono* and *Pspc1*. It shows that while both are expressed in all cell types, *Nono* expression levels throughout hematopoiesis are higher than *Pspc1* (Figure 3B and 3C, about 4-fold). This may explain why the PSPC1 immunostaining was less vivid compared to NONO (Supplementary Figure 2). Furthermore, this data suggests no substantial differences among the examined cell types, with the exception of granulocytes. HSCs have a higher expression of *Nono* and *Pspc1 as* compared to granulocytes by 3.28 and 2.04-fold respectively (*p*<0.01). On the other hand, only *Pspc1* showed higher expression in HSCs compared to GMPs, by 2.32-fold (*p*<0.01). Also, HSCs have a slightly higher mRNA expression for *Nono* and *Pspc1* compared with naïve B-cells by 1.59 and 1.18-fold, respectively (*p*<0.01 and p=0.018), and for marginal-zone B-cells by 1.64 and 1.34-fold for *Nono* and *Pspc1*, respectively (*p*<0.01, both). This RNA expression data is in concordance with the protein staining quantification we performed on the cells stained for NONO (Figure 3A). Nevertheless, the enumeration for the NONO foci done by CellProfiler found no significant differences between the number of foci in the cells that were stained, except for granulocytes which showed reduced number of foci compared to HSCs (Figure 3D, average foci per cell 4.3 to 16.1 respectively, *p*=0.018). Thus, we can see a good agreement between RNA and protein levels. The high standard deviation of CellProfiler analysis might derive at least in part from inherent technical inability to enumerate the foci in the highest microscopic resolution. Nevertheless, microscopic images present a substantial variability of paraspeckle formation, suggesting that along with the significant difference between the population-averages of HSCs and granulocytes, there is truly a substantial variability between cells of the same population with respect to NONO foci numbers.

### *Neat1* expression is enhanced by differentiation *in vitro*

**Figure 4:**
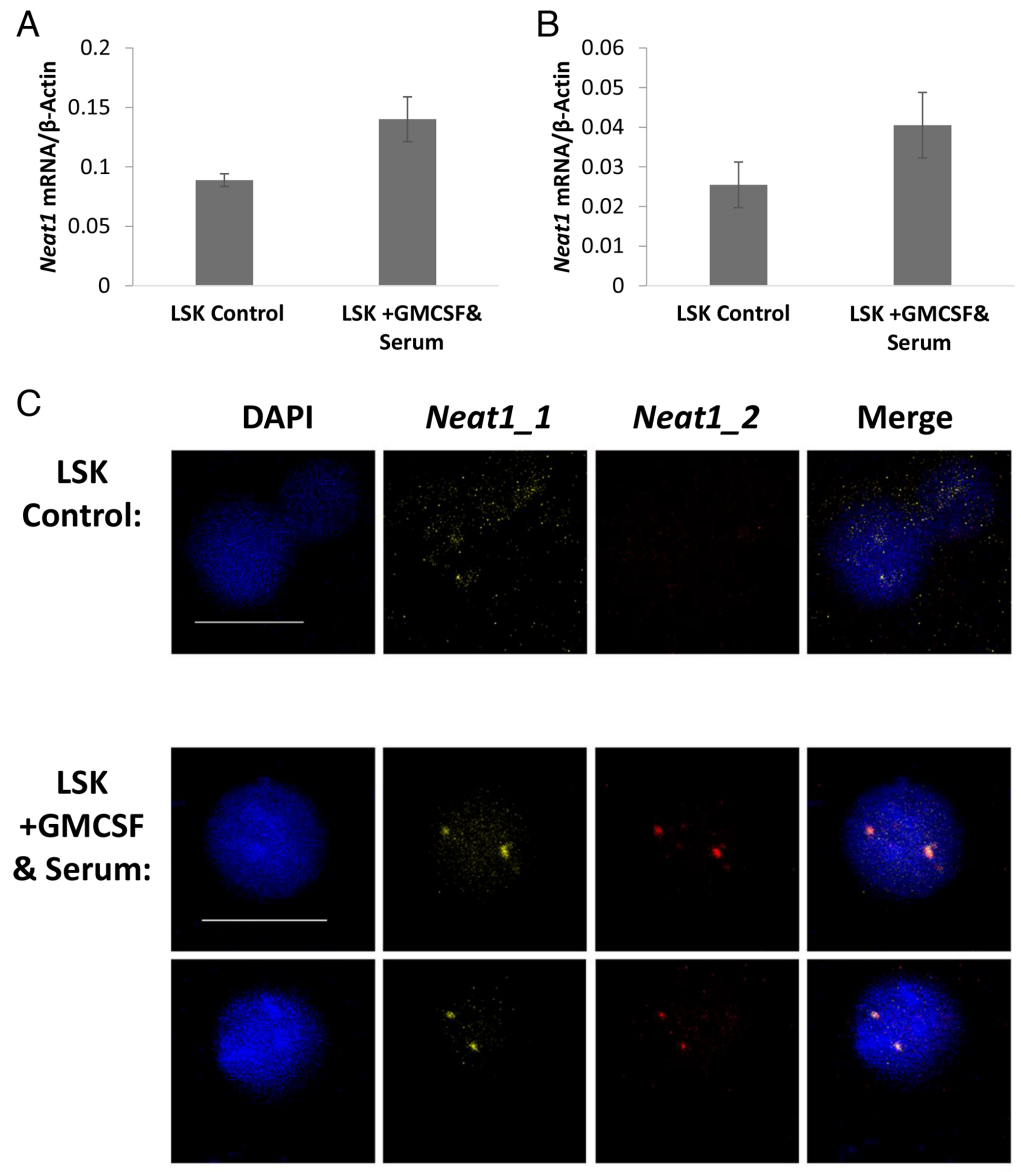
*Neat1* expression is enhanced by differentiation *in vitro* **(A and B)** Expression levels of *Neat1_1* and *Neat1_2* measured by RT-qPCR and shown relative to β-Actin. GMCSF and Serum were added to the medium for the last 96 hours of the culture. Histograms show averages ±SD. **(C)** Confocal microscopy FISH of *Neat1_1* and *Neat1_2*. Scale bar denotes 10 μ.m.

Subsequent to the findings in primary fresh cells, we tested cells in culture. To induce differentiation, cultured primary cells were split into separate wells and while some remained on minimal-media the others were supplemented with GM-CSF and Serum to induce further differentiation. After 96 hours of induction the cells were examined by RNA quantification, FISH, and Immunofluorescence staining. RT-qPCR for *Neat1* isoforms expression was normalized to the housekeeping gene β-Actin. The addition of GM-CSF and Serum to the culture medium increased the RNA expression of *Neat1_1* by 1.56-fold (*p<*0.01) and *Neat1_2* by 1.59-fold (*p=*0.03, Figure 4A and 4B). Similar results were seen in independent experiments (with the total of 5 biological replicates; Supplementary Figure 3A and 3B). FISH detection of *Neat1_1* and *Neat1_2* RNA in the cultured cells yielded bright expression. Microscopic images show that as a consequence of adding GM-CSF and serum to the cultured cells there was an increase in the staining of *Neat1_1* and relatively higher increase of *Neat1_2* (Figure 4C). This comes in concordance with the higher expression levels shown using RT-qPCR. Moreover, there is a better colocalization of *Neat1_1* and *Neat1_2,* suggesting the enhancement of paraspeckles in cultured hematopoietic cells that were pushed towards differentiation. Similar results were visualized for cultured primary MPPs (Supplementary Figure 3), further implying that the induction of differentiation *in-vitro* upregulates *Neat1* expression, and possibly the paraspeckle formation in hematopoietic cells.

### NONO sustains aggregated nuclear expression in cultured cells

**Figure 5:**
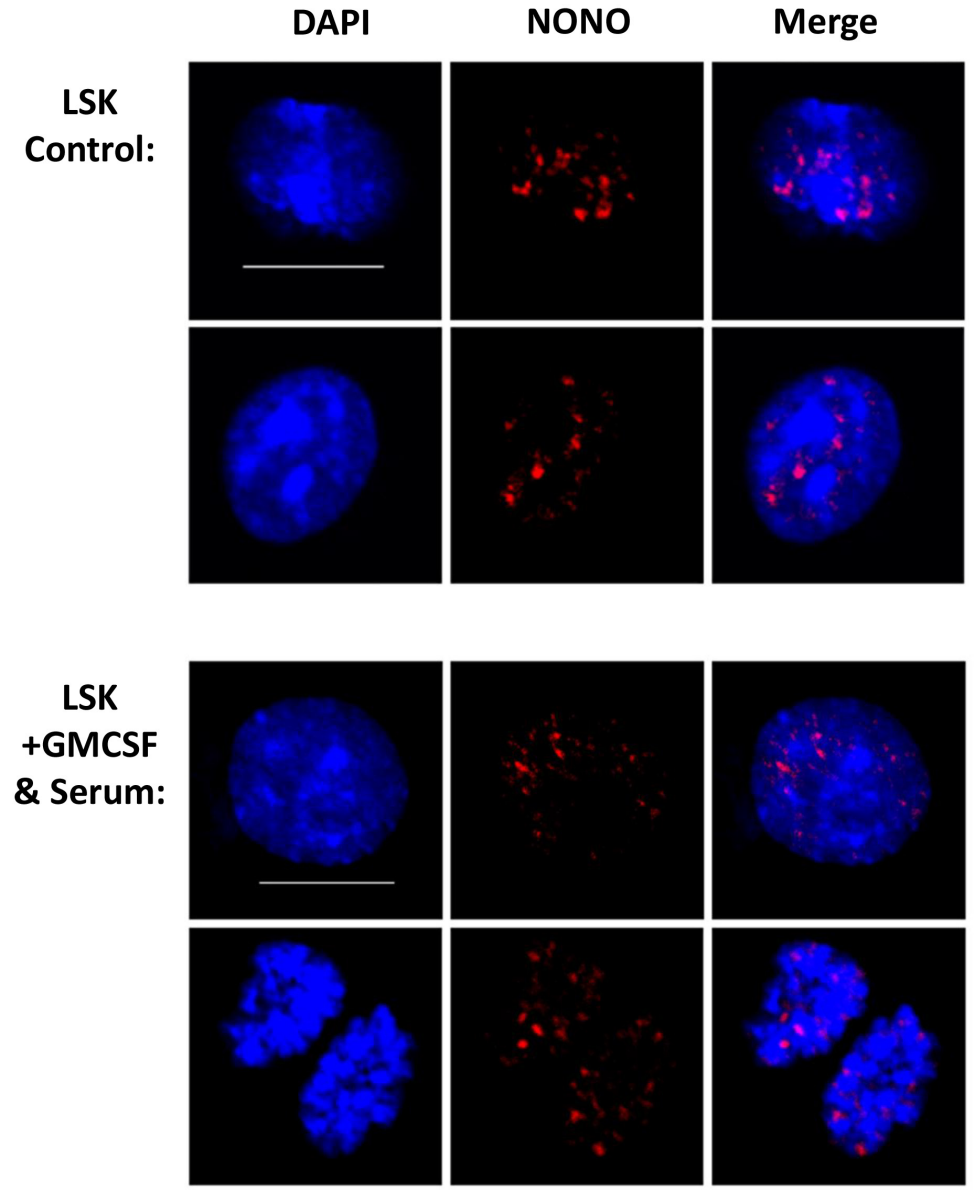
NONO sustains aggregated nuclear expression in cultured cells Confocal microscopy analysis of NONO using Immunofluorescent staining technique for cultured cells. Representative images are shown from independent experiments and total of 5 biological replicates. Scale bar denotes 10 μ.m.

To further visualize paraspeckles in the cultured cells we stained for the NONO protein. The cultured cells did not show any significant difference in the amount, intensity or size of NONO foci (Figure 5 and Supplementary Figure 4). Although there was no difference between cells that were induced into differentiation, cells of both groups sustained nuclear foci integrity, suggesting that the culture conditions are not dismantling paraspeckles. This finding strengthens the claim of presence of these sub-nuclear structures in hematopoietic cells, not only *in vivo* but also *in vitro*. In addition, it further suggests that there is no strict limiting correlation between the paraspeckle proteins and the *Neat1* RNA.

## DISCUSSION

Learning of the expression of *Neat1* and formation of paraspeckles in hematopoietic cells will advance the research in the field of normal hematopoiesis and blood malignancy, considering the data on *Neat1* in other cancer types and the lack of data in the hematopoietic system [24-26]. In this study, we have laid the foundation to elucidating the manner in which *Neat1* and paraspeckles are expressed in hematopoietic stem-cells, progenitors, and differentiated immune cells both *in vivo* and *in vitro*. Our data demonstrate the expression of *Neat1* and visualize paraspeckle formation in HSCs and progenitors. We do identify pronounced downregulation of *Neat1* and paraspeckles proteins in granulocytes, but not in B-cells. These findings will lead for innovative research in the fields of hematopoiesis, stem cell differentiation and in blood cancers.

### *Neat1* and its isoforms in hematopoietic cells

Our data demonstrate that *Neat1* is expressed in HSCs, progenitors and immune cells, by analysis of RNA-seq, independent RT-qPCR and direct FISH visualization. Furthermore, *Neat1* seems to form sharp nuclear foci in these cells, both *in vivo* and *in vitro*. Neat1_1 is highly expressed, quantified at 0.1-fold of β-Actin which is known to be an extremely-highly expressed gene, and is visualized most clearly in all cells. Although Neat1_1 is very profound, Neat1_2 is not expressed as much, and when attempting to visualize it appeared stronger in HSCs and MPPs than in the differentiated cells such as granulocytes or B-cells. This may suggest that when a cell has the potential to proliferate and differentiate it expresses higher levels of *Neat1,* especially *Neat1_2*. This might be of special interest with recent discovery of *NEAT1_2* in pri-miRNA processing [16]. Cells which are terminally differentiated, and post-mitotic (like granulocytes) do not express *Neat1* as much. This is in concordance with the data in the literature concerning the role of *Neat1* and paraspeckles in cell cycle of other cell types [1, 7, 8, 12, 22, 26]. This point is of interest in malignant hematopoietic cells, which may consist of highly-proliferative along with more quiescent cells, and would specifically be of interest regarding Leukemia-Initiating-Cells (LICs, sometimes called “cancer stem cells”). Our data indicate that HSCs, which are deeply quiescent, express *Neat1* at their dormant primary multipotent state.

### Paraspeckles are formed in hematopoietic cells

Paraspeckles are formed in most cell types, with the exception of hESC [11], yet up until now paraspeckles were not visualized in hematopoietic cells. In this study, we show for the first time the foci formed by paraspeckles proteins NONO and PSPC1 in primary hematopoietic cells. We found that *Nono* is highly expressed compared to *Pspc1* at the mRNA level, and the microscopic images suggested similar order with substantially Higher NONO than PSPC1 protein staining (Figure 3A and Supplementary Figure 2). PSPC1 images were generally less-sharp, possibly due to its lower expression levels. Interestingly, enumeration of NONO foci, as a proxy for paraspeckles, found substantial variability among cells (Figure 3D). This is in concordance with the literature as NONO is expressed in foci of various sizes, intensities and number per nucleus in various cells such as Myoblasts, HeLa, NIH 3T3, and other cell lines [1, 7, 22]. Finding paraspeckles protein’s foci is not obvious, because in some cases it was reported that when *Neat1* expression is downregulated they are disrupted, showing diffused staining, suggesting dismantle of the sub-nuclear structure [10]. All the hematopoietic cells tested presented with aggregated NONO and PSPC1 proteins, suggesting no dismantling of paraspeckle foci in these normal primary cells. It was reported that paraspeckles and *Neat1* may change with the cell cycle of other cells [8, 12, 26]. However, finding high levels of *Neat1*, NONO and PSPC1 in HSCs is in contrast with their non-proliferative dormant state. Our current data suggest no correlation of *Neat1* or paraspeckle formation with HSC quiescence. Moreover, we visualized paraspeckles in sub-nuclear areas that are euchromatic, thus their DNA is in a transcription-ready state. These two findings imply that *Neat1* and paraspeckles are fairly abundant in primary quiescent HSCs and active progenitor cells, thus they may have regulatory role on the proliferative potential rather than the acute stage within the cell cycle.

### Paraspeckle foci are sharpened with induced-differentiation ex-vivo

After previous publications regarding *Neat1*’s role in other cell types, and its overexpression during excessive transcription and DNA replication [8-10, 12, 17, 26], we hypothesized that hematopoietic cells pushed towards differentiation would upregulate their expression of *Neat1* and paraspeckle formation. Our RT-qPCR and the images acquired using FISH to visualize *Neat1* were in line with this hypothesis. Thus, it is suggested that *Neat1* may play a role in differentiation of effector cells. Interestingly, the aggregation of paraspeckle proteins at euchromatic regions in the nucleus and their reported role in DNA-breaks in Mouse Embryonic Fibroblasts (MEFs) [27] may further suggest a possible similar function in hematopoietic cells. It would therefore be interesting to further study *Neat1* and paraspeckles in HSCs throughout aging, which is reported to involve the accumulation of DNA-damage and double-strand breaks [28].

## MATERIALS AND METHODS

### RT-qPCR

Experiments were performed according to the local and state ethics committee approval. Cells were extracted from the femur, pelvis, and tibia of wildtype C57BL/6J mice that are housed at the SPF unit of BGU; mice were euthanized using isoflurane and bone marrow cells were obtained by crushing; mononuclear cells were enriched by Histopaque (H1083, Sigma-Aldrich). Cells were stained for Lineage (Ter119, CD11b, GR-1, CD3e, CD4, CD8, and B220), Sca1, cKit, CD34, CD150 and CD48, or for B220, CD19, GR-1 and MAC1; all antibodies from Biolegend, see Supplementary Figure 5 for FACS-gating. Cells were sorted by FACSAriaIII (BD biosciences) and stored in TRIzol (Invitrogen) for RNA, or fixed and stained for FISH and immunohistochemistry. Total of 6 mice in 3 independent experiments were used for RT-qPCRs; triplicates were routinely used for statistical analysis of RT-qPCR. RNA was extracted according to the manufacturer’s protocol, followed by reverse transcription using PrimeScript™ RT-PCR Kit (Takara). qPCR reactions were set with Kappa SYBR Fast qPCR master mix, and run on LightCycler 480 (Roche), using following Primers; Neat1_1 forward: GATCGGGACCCCAGTGACCT Reverse: AGCTTTCCCCAACACCCACA, Neat1_2 forward: GCTCTGGGACCTTCGTGACTCT Reverse: CTGCCTTGGCTTGGAAATGTAA; Actin-beta forward: CTCTGGCTCCTAGCACCATGAAGA Reverse: GTAAAACGCAGCTCAGTAACAGTCCG. PCR program was: Pre-incubation heat to 95°C 5min sec; amplification 45 cycles of 95°C 5sec, 60°C 15sec, 72°C 20sec; melting heat to 95°C 5sec, 65°C 1min and then heat to 97°C by 0.11C/sec.

### Immunohistochemistry

Bone-marrow cells were extracted from femur, tibia and pelvis as above; for primary fresh cells, a total of 9 mice in 3 independent experiments were used, and for cultured cells a total of 5 mice in 2 independent experiments were used. Sorted cells were taken for immunostaining in 96U well Falcon plate. Fixation and permeabilization of cells using “True-Nuclear™ Transcription Factor Buffer Set [CAT:424401, BioLegend]. The anti-mouse H-2Dd clone: 34-2-12 [CAT: 110602 BioLegend] was used for isotype control. Monoclonal Anti-PSPC1 cat#SAB4200503 and monoclonal Anti-NONO cat#SAB4200502 (Sigma). Cells were stained overnight at 4°C, washed twice and fluorescently labeled with secondary Dylight™ 594-conjugated AffiniPure Rat Anti-Mouse IgG [cat# 415-515-166, Jackson ImmunoResearch]. DNA was stained with DAPI (4’,6-Diamidino-2-Phenylindole, Dihydrochloride; 1μg/ml). Cells were mounted on slides and images taken with Olympus Confocal Microscope using Olympus Fluoview FV1000. Quantification of foci was done using the CellProfiler software by Broad Institute [32]. For fresh or for cultured cells about 10 or 30 cells were used for CellProfiler analysis, respectively.

### Fluorescence in situ hybridization (FISH)

Sorted cells (as above) were obtained from a total of 5 mice in 2 independent experiments. FISH was performed in 96U well Falcon plates after Fixation for 10 minutes with 4%PFA in PBS [cat:#15710 Electron Microscopy Science]; Permeabilization for two hours in 70% Ethanol; Hybridization using the two probes overnight at 30°C. Probes were Stellaris® FISH Probe Mouse Neat1 5’ Segment with Quasar® 570 Dye [SMF-3009-1], and Stellaris® FISH Probe Mouse Neat1 Middle Segment with Quasar® 670 Dye [SMF-3010-1, custom-conjugated to enable dual-color detection]. Nuclei were counter-stained using DAPI 1μg/ml. Cells were mounted on slides using GLOX mounting media [contains: Tris pH 8.0, SSC, glucose and nuclease free water] supplemented with 1μL Catalase and 1μL Glucose Oxidase. Cells were observed using Olympus Confocal Microscope and images were taken using Olympus Fluoview FV1000.

### Primary culture

Sorted cells were cultured in BioTraget media (Biological Industries, Israel) supplemented with 10ng/ml of SCF, TPO, IL-3 and Flt3L (Peprotech) as a minimal media that support cell viability *in vitro*. Cells were grown in 96U plates with the media changed twice a week and split according to their growth. Differentiation was further induced by adding GM-CSF (10ng/ml) and Fetal Bovine Serum (10%). Viability was tested microscopically to ensure that cells were in good condition. Cells from individual mice were cultured in parallel to obtain biological replicates, and independent experiments were performed from independent primary sorts, using total of 5 mice in 2 independent experiments.

### Statistical analysis

Results are shown as the mean ±standard deviation. Two-tailed and one-tailed paired Student’s t-tests were used to compare different groups. Statistical *p* < 0.05 was considered significant.

## CONCLUSIONS

The data we present hereby provide the foundation for studying the effect(s) of lncRNA *Neat1* and paraspeckles on blood cancer, including Leukemia, Myeloma or Lymphoma. Other studies showed that *Neat1* may increase proliferation, invasion, Endothelial-to-Mesenchymal-Transition (EMT), and chemo-resistance in multiple cancer types including lung cancer, breast cancer and in renal-cell carcinoma [24-26]. Other lncRNAs were reported to genetically associate and functionally regulate blood cancers [2, 5]. Yet, there was no study that spotlighted *Neat1* and focused on its role in paraspeckle formation in hematologic cancers. Most interestingly, a recent study revealed *Neat1* dysregulation in Multiple Myeloma [29], pioneering its clinical hematological relevance. Strikingly, *Neat1* was recently reported to be directly induced by p53, and to suppress early malignant neoplasm [4, 20]. *Neat1* had been reported to relate with breast cancer, suggesting it as a biomarker [30]. We had searched for hematological cancers survival correlation through SurvExpress [31], finding significant Hazard Risk with some of the Lymphoma and in Multiple Myeloma (data not shown), suggesting further focused interest. Our study offers a base for future research, with solid findings of the normal state and the ability to quantify and visualize *Neat1* and paraspeckle proteins in primary hematopoietic cells. This study demonstrates, for the first time, the consistent expression of *Neat1_1* and the formation of paraspeckles in HSCs, progenitors and effector immune cells.

## SUPPLEMENTARY MATERIALS FIGURES



## Abbreviations

*Neat1*: Nuclear Enriched Abundant Transcript 1
HSCs: hematopoietic stem cells
lncRNAs: long non-coding RNAs
*Menε*: Multiple Endocrine Neoplasia ε
*Menβ*: Multiple Endocrine Neoplasia β
hESC: human embryonic stem cell
MPP: multipotent progenitors
CMP: common myeloid progenitor
GMP: g*ranulocyte-macrophage progenitor*
MEP: megakaryocyte-erythroid progenitor
Gran: granulocyte
CLP: common lymphoid progenitors
FISH: Fluorescence in Situ Hybridization
MEFs: Mouse Embryonic Fibroblasts
GM-CSF: granulocyte-macrophage colony-stimulating factor
EMT: Endothelial-to-Mesenchymal-Transition
RNA-seq: RNA sequencing
RT-qPCR: reverse transcription quantitative polymerase chain reaction
PFA: paraformaldehyde
PBS: phosphate-buffered saline
