# Supplementary material for: Neat1 in hematopoietic stem cells
Source: Oncotarget. 2017 Nov 30;8(65):109575–86. doi: 10.18632/oncotarget.22729 (PMC5752543; doi:10.18632/oncotarget.22729)
Supplement: Supplementary file 1 [file oncotarget-08-109575-s001.pdf]

Neat1 in hematopoietic stem cells

SUPPLEMENTARY MATERIALS

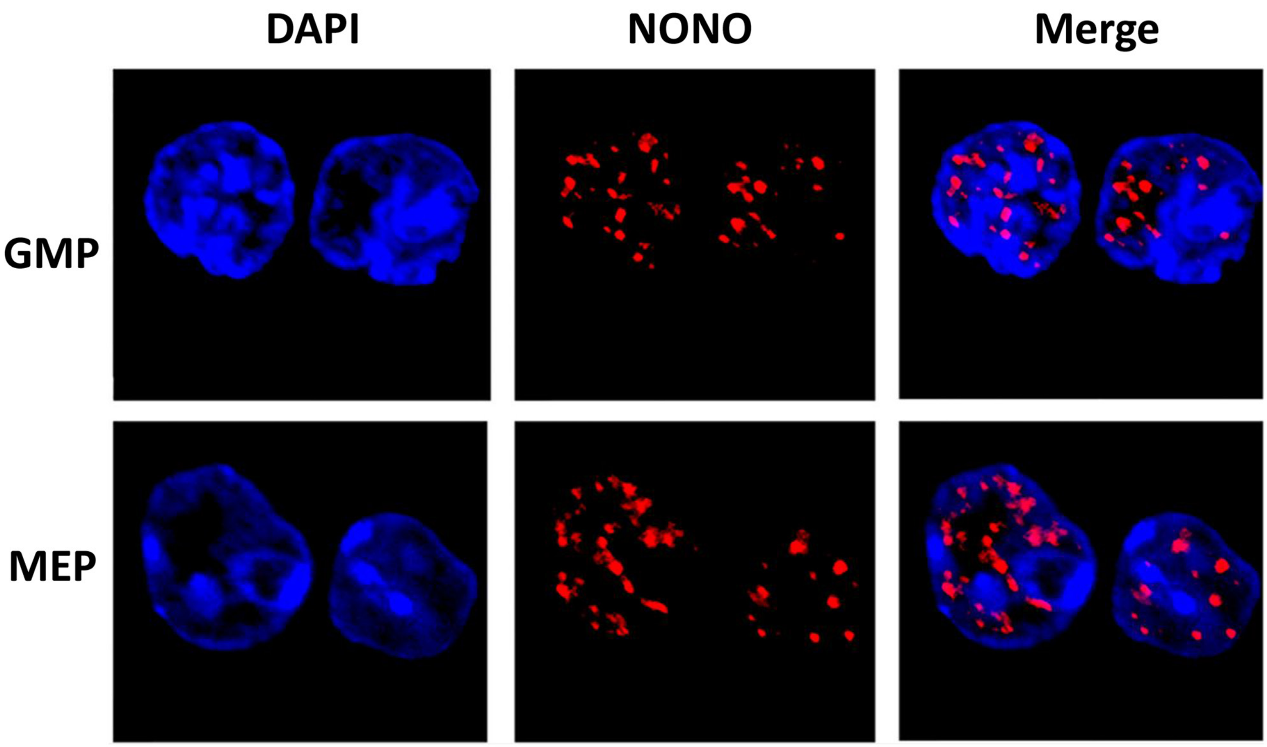

Supplementary Figure 1: NONO Foci are concentrated in euchromatin areas of the nucleus. Confocal microscopy analysis of NONO, a paraspeckle protein, using Immunofluorescent staining technique, in representative primary cell types GMP and MEP, enlarged. NONO (red) is superimposed over DNA stained with DAPI (blue).

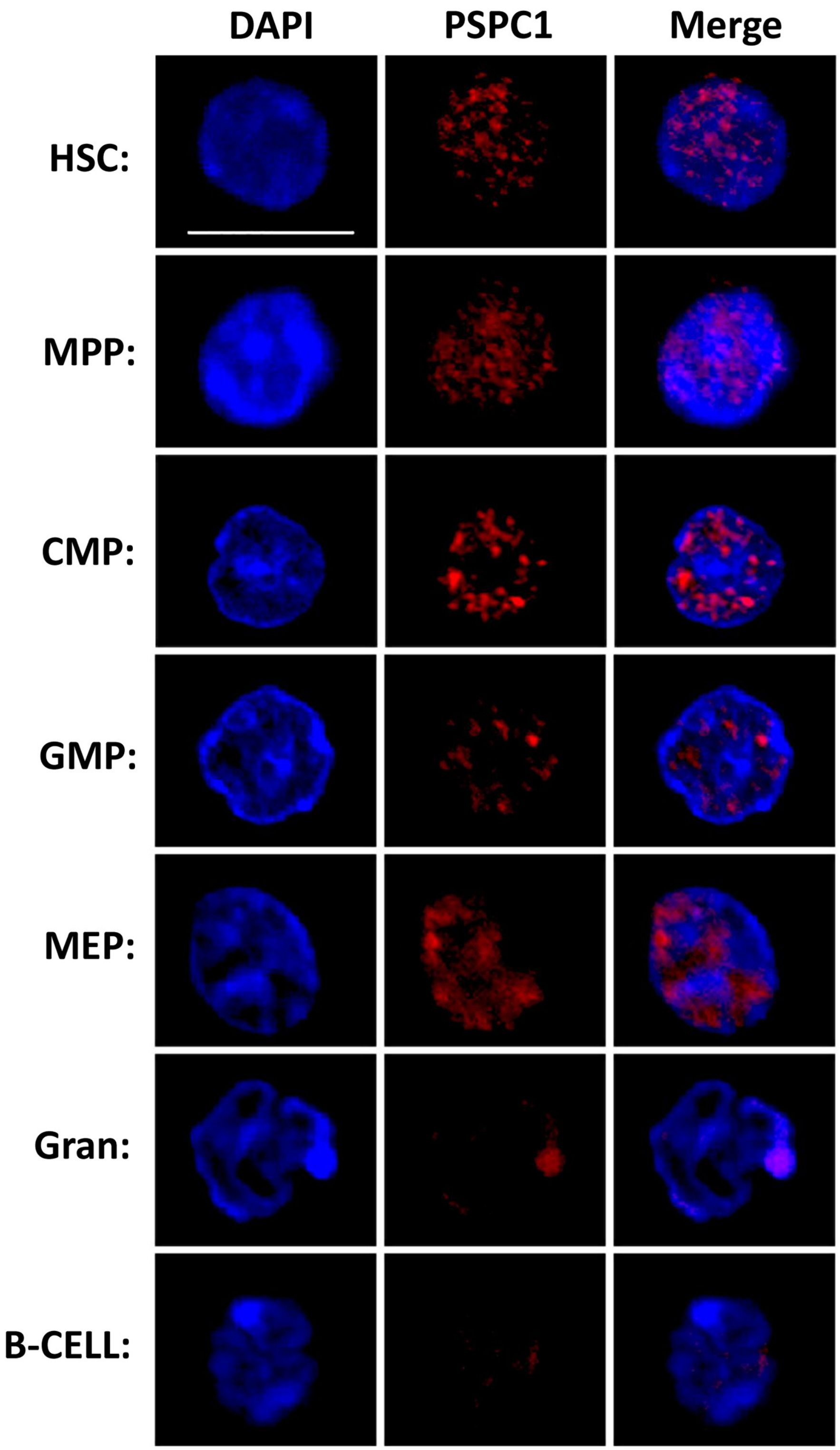

Supplementary Figure 2: Paraspeckle protein PSPC1 presents aggregated clusters in hematopoietic cell's nuclei. Confocal microscopy analysis of PSPC1, a paraspeckle protein, using an immunofluorescent staining technique, in representative primary cell types: HSC, MPP, CMP, GMP, MEP, granulocyte, B-Cell. PSPC1 (red) is superimposed over DAPI (blue). Scale bar denotes 10 µ.m.

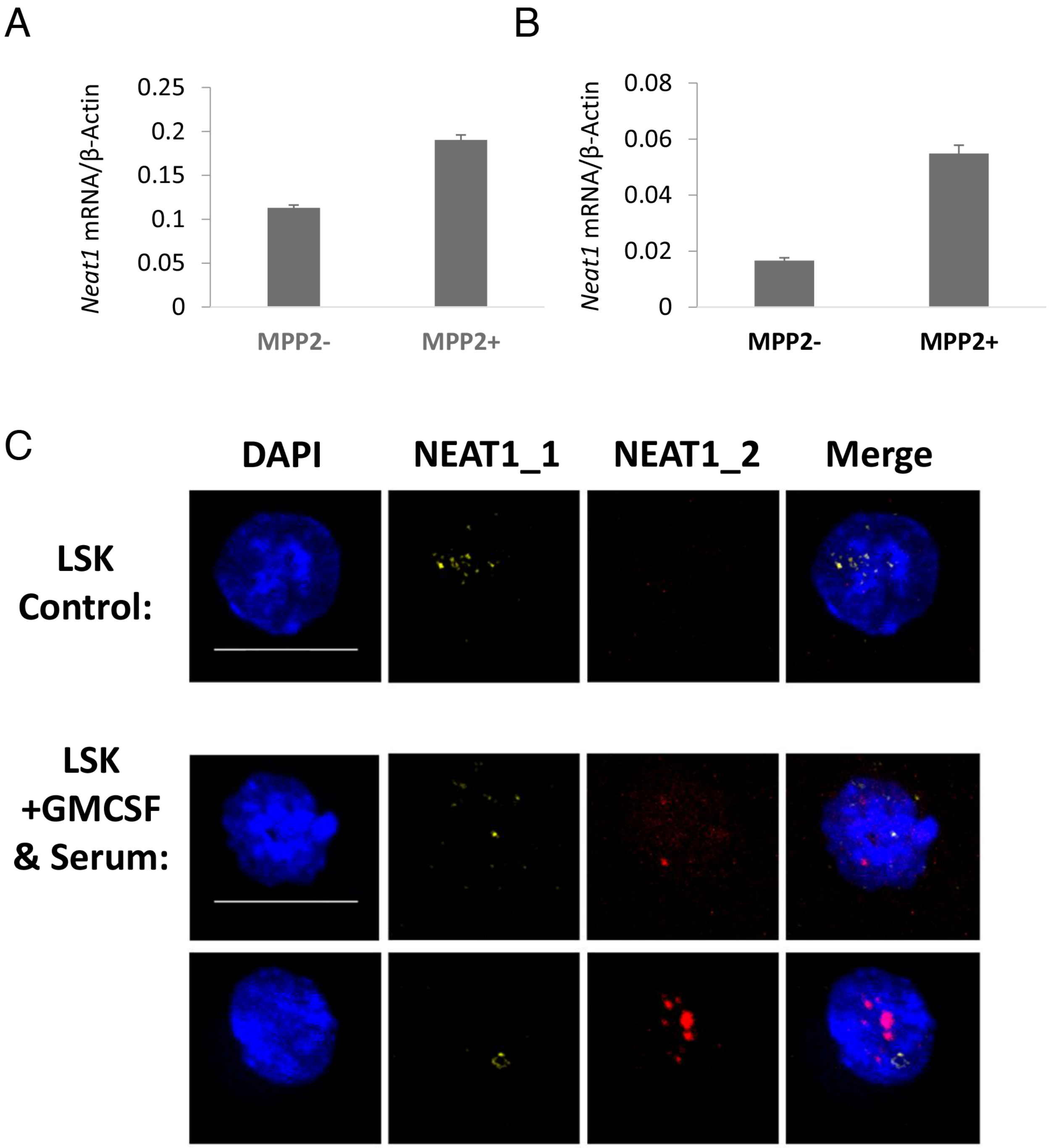

Supplementary Figure 3: *Neat1* expression is enhanced by differentiation *in vitro*. (A and B) Expression levels of *Neat1_1* and *Neat1_2* measured by RT-qPCR and shown relative to β-Actin. GM-CSF and Serum were added to the medium for the last 96 hours of the culture. Histograms show averages ±SD. **(C)** Confocal microscopy FISH of *Neat1_1* and *Neat1_2*. Scale bar denotes 10 µ.m.

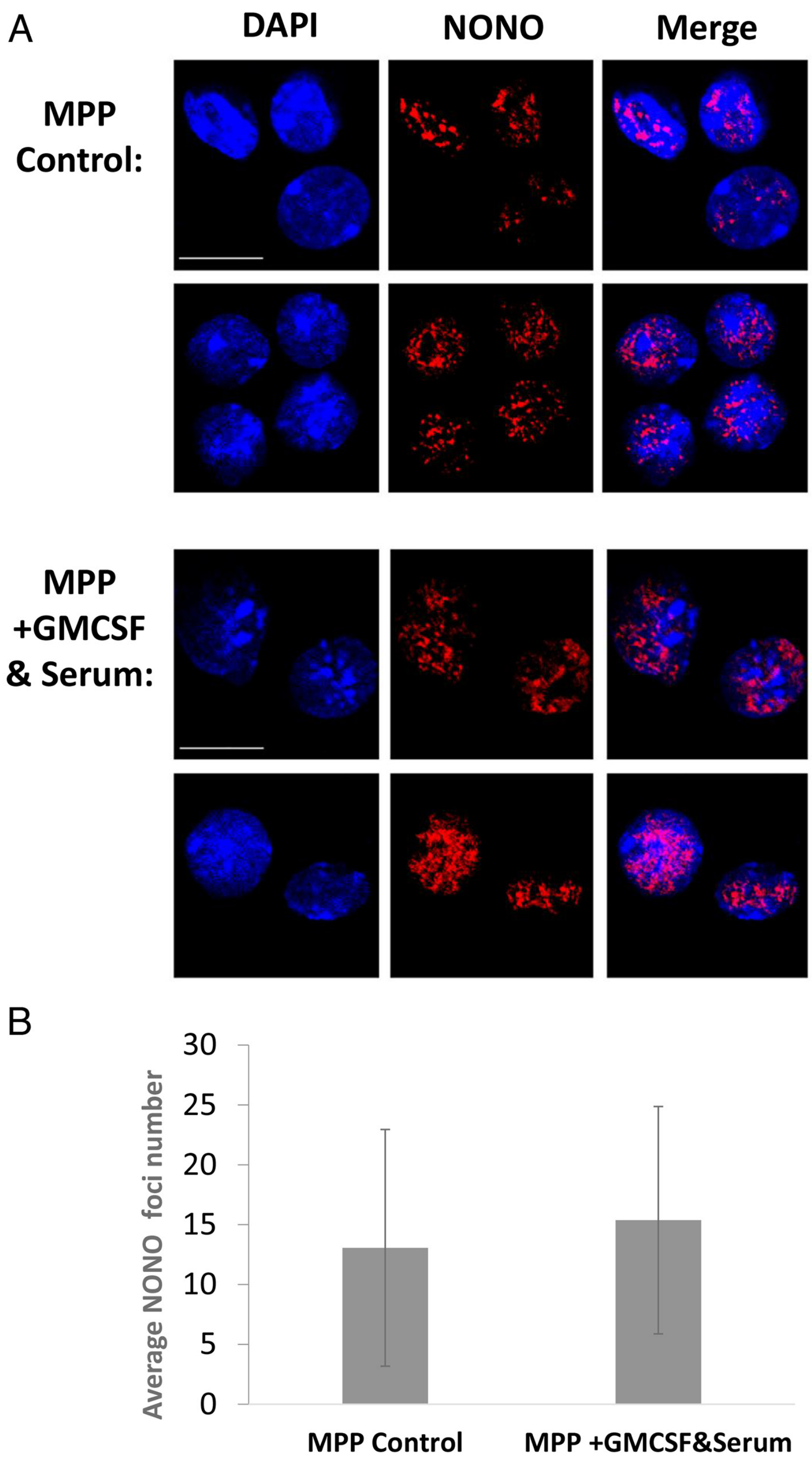

Supplementary Figure 4: NONO sustains aggregated nuclear expression in cultured cells. **(A)** Confocal microscopy immunofluorescent analysis of NONO. Representative images are shown from independent experiments and a total of 5 biological replicates. Scale bar denotes 10 µ.m. **(B)** Quantification of the NONO foci number in cultured MPPs with and without the addition of GM-CSF and serum, by CellProfiler software. Histograms show averages ±SD.

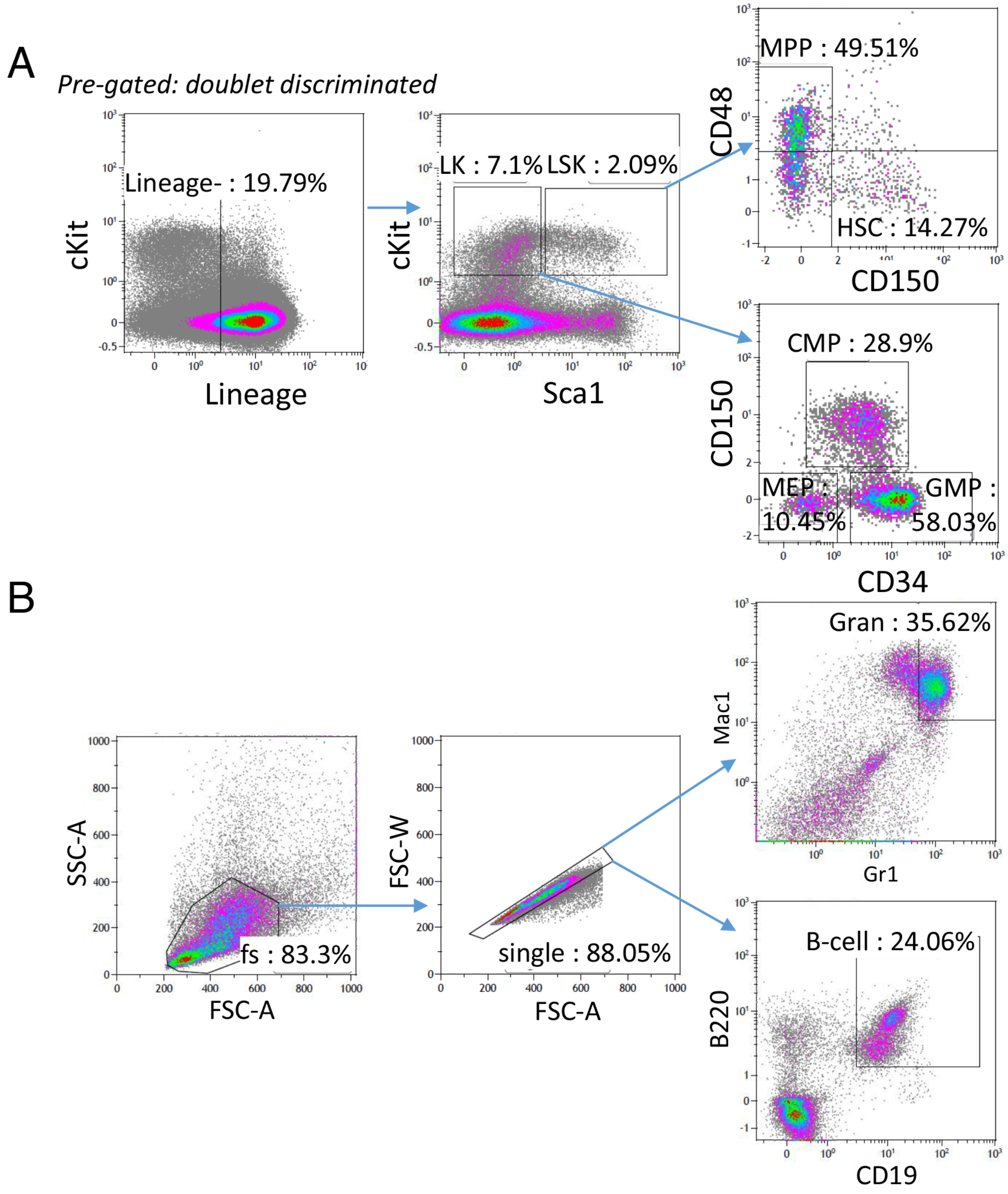

Supplementary Figure 5: FACS gating for the isolation of primary HSCs, progenitors and effector immune cells. (A) Gating strategies used to isolate HSC (Linage$^-$, Sca1$^+$, cKit$^+$, CD48$^-$, CD150$^+$), MPP (Linage$^-$, Sca1$^+$, cKit$^+$, CD48$^+$, CD150$^-$), CMP (Linage$^-$, Sca1$^-$, cKit$^+$, CD34^{med}, CD150$^+$), GMP (Linage$^-$, Sca1$^-$, cKit$^+$, CD34^{high}, CD150$^-$), MEP, (Linage$^-$, Sca1$^-$, cKit$^+$, CD34$^-$, CD150$^-$). **(B)** Gating strategies used to isolate granulocytes (Mac1$^+$, Gr1$^+$), and B-Cells (B220$^+$, CD19$^+$).
